# William Tuke, the Founder of the York Retreat
*The particulars of this sketch have been furnished us by Dr. D. H. Tuke, of York, the great-grandson of the founder of the Retreat.—Ed. P. J.


**Published:** 1855-10-01

**Authors:** 


### WILLIAM TUKE, THE FOUNDER OF THE YORK RETREAT.*

William Tuke was born at York in the year 1732. His ancestors had resided for many generations in that city, and were descended, in all probability, from a family long settled in the south of Yorkshire, and the adjacent county of Nottingham.

The York branch early suffered for Nonconformity; the great-grandfather and namesake of the subject of the present sketch having advocated the doctrines of the Society of Friends soon after its rise, and submitted to imprisonment, and the loss of property, on account of his religious opinions.

* The particulars of this sketch have been furnished us by Dr. D. H. Tuke, of York, the great-grandson of the founder of the Retreat.—Ed. P. J.

When a boy, William had well nigh lost his life by a fall from a tree, which he had climbed in search of a bird's nest. An eminent surgeon was sent for, however, who found the skull fractured, and performed the operation of trephining. Though thus apparently fond of bird-nesting, he was not an idle school-boy, and, in addition to the acquirement of rudimentary knowledge, made considerable progress in the Latin language, retaining in advanced age a lively recollection of some passages of Virgil's "Georgics." After being at a day-school, he enjoyed the advantages of a boarding-school, and was for some time placed by his father under the tutorship of a clergyman.

He married at the age of twenty, and had by his first wife five children, the eldest of whom, Henry, co-operated with him in his exertions relative to the Retreat.

In 1765, he took a second wife, by whom he had three children. For the last twenty-eight years of his life he was a widower.

During the greater part of his life, William Tuke was engaged in mercantile pursuits, but was able to devote a large share of his time to objects of a public and philanthropic character. He is thus described in an obituary published in the public papers of the period:—"There will scarcely be found an instance of any useful or benevolent undertaking, within the proper scope of his exertions, which did not partake of his support, not merely in a pecuniary way, if that were needed, but (which is more important) in personal attention. We admire in many excellent characters an ardour amounting to enthusiasm which attaches them, almost exclusively, to some one favourite object; but William Tuke was a philanthropist of all work. Liberal of his time and labour, where-ever these could be brought into use, exemplary in the punctuality of his attendance and in his adherence to the business in hand, and clear in his conceptions of its nature and bearings, he was on all occasions of this nature an able and a welcome coadjutor. In short, he was one of those rare characters who 'are never weary in well doing,' and who accomplish it in the most efficient way."

But while the objects of William Tuke's benevolent exertions were thus various, the subject which undoubtedly most occupied his time and attention, and for which his name will be chiefly remembered, was the establishment of the Retreat at York.

In the year 1791, a lady, a member of the Society of Friends, was placed in the old York Asylum. Her friends, who resided at a distance, requested some of their acquaintance living in the city to pay her a visit. They accordingly went to the Asylum for this purpose, but their request was refused. Very shortly after, the patient died, a circumstance which, in connexion with the conduct of the asylum authorities altogether, excited considerable suspicion, and led William Tuke to feel very strongly the want of an institution for the insane, in the management of which secrecy should be wholly done away with, and which the friends of the patients might therefore regard with confidence. Having a clear perception of a want, he was not the man to remain inactive. It appeared to him that this want might be supplied, and his idea carried out into practice, by a Society which had already exerted itself on behalf of other suffering and neglected classes of the human race. It seemed but fitting to appeal to the friends of the slave and the prisoner, for aid on behalf of those who were incarcerated in loathsome cells, for no other crime than that they had lost their reason.

Accordingly, in the spring of the year 1792, William Tuke made the memorable proposition to a meeting of the Society of Friends held in York, that it should have an institution under its own control, for the care and proper treatment of those who "*laboured under that most afflictive dispensation—the loss of reason.*"

But the proposition was far from meeting, in the first instance, with a cordial response. Some of the speakers denied the want of any such institution; others maintained that it was entirely out of the province of such an assembly to enter into a consideration of the subject; and the greater part manifested (what might naturally have been expected) little acquaintance either with the extent to which insanity existed, or with the actual condition of the insane. A small number, however, including his eldest son, and the well-known grammarian, Lindley Murray, warmly seconded the proposal. At the subsequent conferences on the subject much fresh evidence, which had been collected, was earnestly put forward, and at length the non-contents were satisfied, and allowed the following resolution to be carried:—"*That in case proper encouragement be given, ground be purchased, and a building be erected sufficient to accommodate thirty patients,* in an airy situation, and at as short a distance from York as may be, so as to have the privilege of retirement; and that there be a few acres for keeping cows, and for garden ground for the family, which will afford scope for the patients to take exercise when that may be prudent and suitable*"—a resolution which indicates, very clearly, the enlightened benevolence of its authors. This was also evinced by the name proposed for the establishment—"The Retreat"—by which it was "intended to convey the idea of what such an institution should be, namely, a place in which the unhappy might obtain a refuge; a quiet haven in which the shattered bark might find the means of reparation, or of safety."

A circumstance may here be related which is of interest, inasmuch as it materially strengthened William Tuke's endeavours to ameliorate the condition of the insane. When turning his attention to the subject, he visited St. Luke's Hospital, in the hope of obtaining information, but was afresh impressed with the necessity of some such institution as the Retreat, by what he witnessed there. He saw the patients miserably coerced, not from intentional cruelty, but from a conviction of the superiority of such a course of treatment over any other. Among them was a young woman, whose condition especially arrested his attention, and excited his compassion. She was without clothing, and lay in some loose, dirty straw, chained to the wall. The form of this unhappy patient haunted him afterwards, and redoubled his exertions, until his plans were carried into practical effect.†

The success of the best plans depends, however, upon the execution. "He had hoped to have found among his numerous friends some one (we may say like himself) devoted to the good of man, and who having leisure for such an engagement would have taken upon him the voluntary and gratuitous superintendence of the establishment. Such a superintendent he thought he had found in a brother-in-law, who had just retired from medical practice, and who entered into the project with much interest. He consented to take the office, at least temporarily, and was in the institution at its opening; but in about two months he was removed by death. The founder looked around among his friends for a suitable successor, but not finding one ready for the engagement, he agreed to undertake the office himself till a substitute should be found, and for nearly twelve months he had the immediate management of the young establishment upon him. This opportunity for close observation, confirmed his estimate of the new Institution, and enlarged his hopes of what might be done, in the improvement of the management of the insane. He only wanted efficient resident agents. Ultimately, the right man was found in the person of George Jepson.

* It was soon found necessary to provide for a larger number; there are at the present time 114 patients in the Retreat, a considerable number of whom are not members of the Society of Friends.

† A few years passed away, and she became an inmate of the York Retreat, and we find its founder observing, in a letter written to an intimate friend, that "she has got settled, and appears more comfortable than at St. Luke's."

It was, indeed, a rare concurrence of circumstances which brought together two minds, one so capable to design wisely and largely, and the other so admirably fitted to carry such designs into execution.

The two men, though exceedingly different, were one in an earnest love to God and man—in disinterestedness and decision of character; and, therefore, in a steady, conscientious perseverance, which worked onward wherever truth and duty led. Both of them had a strong faith in the dictates of an enlightened conscience, and in the perfect wisdom and love which direct every law of human duty. He was of course initiated into the duties of his office by William Tuke, who long continued his parental care of the institution, and may be said for a considerable time to have been virtually manager-in-chief. When the new superintendent had fully obtained his esteem and confidence, he still continued his vigilant oversight, and, as treasurer, regularly conducted the financial and some other parts of the correspondence of the institution, till the decay of his sight obliged him, in his eighty-eighth year, to close his long and gratuitous services.*

He had the satisfaction of witnessing the complete success of the experiment, not only in regard to its direct and primary object, but also indirectly by its influence upon other asylums for the insane.

He lived to take an active part in the exposure of the cruelties† enacted at the very asylum, the conduct of whose authorities had led, twenty-three years before, to the projection of the Retreat, the published "Description" of which, by his grandson,‡ was the immediate occasion of the controversy which terminated in an entire reformation in the management of the old York Asylum.§

In regard to the views entertained by William Tuke and his fellow-labourers respecting the use of personal restraint, it may be well to state, that while they from the first eschewed the use of chains, hobbles, and other harsh instruments of coercion, and in so doing evinced indubitable boldness and humanity, departing as they did from the treatment advocated and pursued by the highest authorities, they never theorized upon or systematized the subject. They decided conscientiously, and with remarkable judgment, in each individual case as it presented itself, acting rather in accordance with what appeared to them right and reasonable, than following the doctrines of the schools. Although carrying on this experiment contemporaneously with

* "Review of the Early History of the Retreat." 1846.

† "I am not quite satisfied that the past abuses of the Asylum should be referred to in vague and indefinite terms—fraus latet in generalibus—nor is it enough to say that squalidity, filth and rags, were in too many instances predominant—or that the means of occupation, amusement, or exercise, scarce existed, for that a hundred patients might, in fine weather, be once a day driven for an hour into a small area, deserves none of these names, or that the cell was fitted to its wretched inhabitants, without light, without air, soaked in urine, and besmeared with ordure."—S. W. Nicoll, Esq. 1814.

‡ "What strenuous efforts fruitlessly combined to accomplish, a little volume, in which this asylum was scarcely mentioned, has at once achieved. I hardly need name Mr. Samuel Tuke's account of the Retreat. Had this interesting work opened the eyes of the old governors, the wonder would have been less ; instead of opening, it closed their eyes ; the more there was to be seen, the less they would see ; the more there was to be done, the less they would do. Mr. Tuke's work, operating on a suspicious and irritable mind, produced the letters signed *Evigilator*, the public attention became roused, doubts and surmises were started. Either confident in right, or daring in wrong, a general challenge was given, that challenge was answered, with what results it is needless to add."—*Vide* "Collection of Papers respecting the York Lunatic Asylum." By S. W. Nicoll, Esq. 1816.

§ The name of Godfrey Higgins, Esq., must never be forgotten in connexion with this subject. What he said of the founder of the Retreat, that "he had reared a monument of goodness to himself, *ære perennius*," may justly be applied to him.

Pinel, they were totally unconscious of the success attending his labours, and had not therefore the advantage of his example.

We need not be surprised that, animated by the same motives, they should in both cases arrive at the same result, namely, the superiority of kindness and judicious treatment over chains and stripes; but in neither instance was the modern doctrine of non-restraint, as set forth by its supporters, asserted; at the same time no one can doubt that then there commenced, in that marked amelioration of the condition of the insane, the real application of those humane principles of treatment, which have in later times led to still more striking results, whether they be found among those who consider "that the use of restraint is never necessary, never justifiable, and always injurious in all cases of lunacy whatever," or among those who only resort to it, as "a necessary evil," in most exceptional cases. Considerable investigation into the early practice pursued at the Retreat induces us to think that the amount of restraint employed was remarkably small, and fully justifies the general description given of it by Dr. Conolly, when he says, "Certainly, restraint was not altogether abolished by them [the early managers of the Retreat], but they undoubtedly began the new system of treatment in this country, and the restraints they did continue to resort to were of the mildest kind."

William Tuke enjoyed the full possession of his mental faculties up to within a week of his death, in 1822; and although blind for several years previously, continued to pursue his active and useful life. Many years before his death, he had occasion to consult the well known Dr. Willan, who singularly enough made the observation, on placing his finger on his wrist,—"There is a pulse which will beat till ninety!"—and so it proved. He was seized while at dinner with a paralytic attack, and for the few following days of his life was more or less delirious. During conscious intervals, however, he was able to converse with those around him, but he was ever a man of few words, and said little more than that he wished to be perfectly quiet; and with a message of affectionate remembrance to the matron of the "Appendage" of the Retreat (which were his last words), he quietly passed away.

He reposes in the same ground where John Woolman, the friend of the slave, is laid; and side by side with Lindley Murray, to whom he was so intimately attached during life—a friendship in unison with the motto on his seal, "Fortior leone amicitia."

A few words may be added to this brief memoir, in regard to his person and character.

"In person," writes a cotemporary, "William Tuke hardly reached the middle size, but was erect, portly, and of a firm step. He had a noble forehead, an eagle eye, a commanding voice, and his mien was dignified and patriarchal. In politics," he adds, "he was invariably Tory, or, as it is now understood, 'a stanch King's man.'

"At the great election of 1807, he spoke from the hustings, in favour of the Hon. Henry Lascelles. A patron of the Bible Society, he attended all its meetings, liberally contributed to its funds, and often edified the members by the weight of his remarks. That saying, 'Crescit amor nummi, quantum ipsa pecunia crescit,' was not verified in his example, for he certainly was one of the most disinterested of men."*

"An object," writes one who knew him well, "which once seriously engaged his attention, he seldom abandoned, being neither depressed by disappointment nor elated by success; but if circumstances proved untoward in the outset, he could wait with patience the favourable moment, and then pursue his object with all the energies of his mind. It was this complete self-government, united with good judgment and unwearied application, which formed the secret of his success. The faculty of mind, which perhaps most distin-

* "Yorkshire Observer." 1822.

guished him, was observation. Scarcely any object escaped his attention, and he had an invaluable stock of facts ready to illustrate almost every occasion. On subjects at all within the sphere of his occupations and engagements, his knowledge may be said to have been profound, for he could not rest in a superficial acquaintance with subjects which came before him. His countenance was the very picture of *strength*. His words were of the same character—though few, they were always effective. During the latter part of his life, there was a great mellowing of what might be called the stern features of his character, and increased condescension and gentleness. He might be often seen with his great grandchildren playing upon his knee, and examining with childish curiosity the indentation on his head, caused by the accident which befell him when a boy."

It would be easy to enlarge upon the traits of William Tuke's character, and to illustrate the expansive benevolence of his heart, by referring to the many objects of a general and local nature which he originated or supported; but it does not fall within the purpose of the present sketch to enter further into detail. Nor would it befit the entire simplicity of his own character, to load his memory with eulogistic expressions; but in regard to his exertions on behalf of the Insane, and of those who co-operated with him, we may say in conclusion that, "although in this engagement they thought not of fame, and pursued their admirable course with a simplicity, almost amounting to unconsciousness of what they were accomplishing, we trust we do not contravene their noble spirit, in having made them, though dead, to speak, by the holding up of their pious example, to ourselves and others."*

---

### RESEARCHES INTO THE FUNCTIONS OF THE BRAIN.

* "Review of the Early History of the Retreat." 1846.

